# Oral propranolol combined with topical timolol for compound infantile hemangiomas: a retrospective study

**DOI:** 10.1038/srep19765

**Published:** 2016-01-28

**Authors:** Jing Ge, Jiawei Zheng, Ling Zhang, Weien Yuan, Haiguang Zhao

**Affiliations:** 1 Department of Oromaxillofacial Head and Neck Surgery, Shanghai Ninth People’s Hospital, College of Stomatology, Shanghai Jiao Tong University School of Medicine, Shanghai Key Laboratory of Stomatology, Shanghai, 200000, China; 2 School of Pharmacy, Shanghai Jiao Tong University, Shanghai, 200000, China; 3 Department of Vascular Surgery, Shanghai Ninth People’s Hospital, College of Stomatology, Shanghai Jiao Tong University School of Medicine, Shanghai Key Laboratory of Stomatology, Shanghai, 200000, China

## Abstract

Compound infantile hemangiomas (IHs) are problematic and usually require intervention. This retrospective study aimed to introduce a combined therapy of oral propranolol and topical timolol, and evaluate its efficacy and safety. Eighty-nine infants with compound IHs were treated with oral propranolol 2 mg/kg/day divided 2 times per day and timolol maleate 0.5% gel 3 times per day, for at least 3 months. Two observers evaluated the hemangioma independently at 0, 1, 3, 6, 9 months after the initiation of treatment. Changes in the hemangioma score values were evaluated using paired t test. Rebound growth and adverse effects were recorded. After treatment was completed, this combined therapy achieved clinical response in 100% of the patients (89/89). Significant positive effects were demonstrated at 1, 3, 6 months (p < 0.001), but not obvious after 6 months (p = 0.06). The response of IHs to the therapy was depending on the age at initial treatment. The average treatment duration was 6.48 (5.77–7.19) months. One patient (1.1%) relapsed after cessation of 6-month treatment, and 7 children (7.8%) developed side effects. Our study suggested that oral propranolol combined with topical timolol treatment is very effective and well-tolerated for compound IHs, which can be used as a first line treatment.

Infantile hemangiomas (IHs), the benign tumours of endothelial cells, is characterized by an initial proliferation during infancy followed by spontaneous involution over the following 5–10 years, often leaving fibro-fatty residues, atrophic scarring, or telangiectasia. The incidence varies from 4% to 10% of infants^1^,^2^,^3^. Problematic hemangiomas occur when they are accompanied with painful persistent skin ulceration, rapid growth, disfigurement, or compromise of normal function or cosmetic development. Common locations for problematic hemangiomas include the periorbital, oropharyngeal, preauricular, or parotid regions. These hemangiomas require early and effective treatment to prevent permanent sequelae. According to its depth of involvement, IH can be classified as superficial, deep, and compound. Superficial IH originates from papillary dermis and presents as bright red macular or papular mass. Deep IH originates from reticular dermis or subcutaneous tissues and appears as bluish or relatively colorless mass. Compound IH is a combination of both superficial and deep components.

Compound IH was reported being treated with oral propranolol, long-pulse pulsed dye laser or intralesional corticosteroid treatment in previously published studies, and the response rate ranged in 66.7%–100%^4^,^5^,^6^,^7^. However, corticosteroids have potential side effects and unknown long-term safety, and long-pulse pulsed dye laser was effective to early hemangiomas only^6^. Hemangeol (propranolol hydrochloride) was FDA approved in the USA on March 17th, 2014 and marketed as the first and only FDA-approved treatment for proliferating IH requiring systemic therapy. However, approximately 13.7% of patients treated with oral propranolol were reported to experience systemic adverse effects^8^. Recently, locally administered timolol was proved as an intervention with acceptable efficacy and lower incidence of adverse effects for superficial IHs, but it has barely no effect on subcutaneous components^9^,^10^. Oral propranolol combined with topical timolol treatment, targeting at deep and superficial component of compound IHs respectively, may maintain clinical efficacy while avoid systemic adverse effects. However, medical literatures about this combined therapy are very limited, and no consensus exists about the proper way to use and monitor this therapy in infants with compound IHs.

The purpose of this retrospective study was to evaluate the efficacy and safety of oral propranolol combined with topical timolol treatment in 89 children with compound IH.

## Material and Methods

A retrospective study was designed and implemented in a tertiary comprehensive hospital in Shanghai, PR China. The study population was composed of all consecutive patients who required treatment of infantile hemangiomas from January 2014 through May 2014. To be included in the study sample, patients must meet the following inclusion criteria: compound hemangiomas with imminent undesirable functional or cosmetic outcomes if left untreated. The exclusion criteria was that the patients have contraindication of ß-blockers, including bronchial asthma, heart failure, sinus bradycardia, hypoglycaemia, hypotension, heart block and known allergy to ß-blockers. Patients with ulcerated or mucosal IHs were also excluded from the study population. Consent to treatment and documentation of the disease response were received from all parents whose infants participated in the present study. The retrospective study followed the tenets of the Declaration of Helsinki for research involving human subjects, informed consent was obtained from all participants, and the study was critically reviewed and approved by the institutional review board of Shanghai Ninth People’s Hospital. The methods were carried out in accordance with the approved guidelines of *SCIENTIFIC REPORTS*.

### Evaluations before and during treatment

Before treatment, all patients underwent a thorough history taking and physical examination, including clinical examination, ultrasound investigation, echocardiography, blood pressure and blood glucose measurements. The patients were scheduled to revisit at 1, 3, 6, 9 months after the initiation of treatment for scoring of IHs, physical examination (including blood pressure, heart rate and blood glucose measurements), measuring of body weight for dosage adjustment, and recording of adverse effects, until the end of treatment.

### Dosage and duration

All patients were treated with oral propranolol and topical timolol, a combination of oral and topical nonselective ß-blockers. The dosage of propranolol (Propranolol Hydrochloride Tablet, Changzhou Kangpu Pharmaceutical Co., Ltd, Jiangsu Province, China) was 2 mg/kg/day divided 2 times daily, meanwhile timolol maleate 0.5% gel (School of Pharmacy, Shanghai Jiao Tong University, China) was applied evenly on the surface of tumours 3 times daily (the dosage was depending on the IH’s surface area). This dosage was maintained during the entire period of the study. The objective of treatment was to inhibit further growth and induce complete regression of the lesions. Treatment was continued until the objective goals were obtained or no further improvement could be achieved. Then the medication of oral propranolol was tapered by decreasing to one half dose for 2 weeks followed by one quarter dose for 2 weeks, and then discontinued. The treatment of topical timolol was ceased when there was no improvement of the superficial component of IH.

### Hemangioma score system

Until now, no standardized or validated method exists for outcomes measurement of hemangiomas. Because the goal of treatment is reducing functional and cosmetic impairment, the semi-quantitative hemangioma score system may be sufficient to meet these goals (Table 1)^11^. Two senior oral and maxillofacial surgeons independently evaluated all patients by determining their hemangioma score at each follow-up visit. For the patient with more than 1 cutaneous IHs, the mean value of each hemangioma score was employed. The mean of the two independent measurements was used for data analysis. At each clinical follow-up visit, the hemangiomas were given a score from 0 to 15 to evaluate the activity and severity. The score system consists of 5 components: color, surface consistency, firmness, depth by ultrasound and organ involvement. Because an organ involvement has the largest medical relevance, this component is the one with the strongest score. The purpose of the score was to monitor and compare the hemangioma from visit to visit individually and from patient to patient. Reducing in hemangioma score was graded as none (0%), minimal (<25%), fair (≥25% and <50%), moderate (≥50% and <75%), near complete (≥75% and <100%) or complete (100%) resolution.

### Statistical analysis

Patient demographics, hemangioma characteristics, indications for treatment were presented as descriptive statistics. The change in the hemangioma score values of the IHs was evaluated using paired t test, comparing the different follow-up visits. All statistical tests were 2-tailed, and α was set at 0.05. Intra-observer reliability was evaluated using the intra-class correlation coefficient (ICC) by standard statistical software packages (SPSS, version17.0, Chicago). A ICC value <0.40 was considered poor agreement, 0.40–0.60 was fair agreement, 0.61–0.80 was good agreement and >0.80 was excellent agreement.

## Results

Patients’ demographic data and were summarized in Table 2. There were 89 children in the study, ranging in age from 1 month to 1.5 years. The mean age was 4.96 ± 4.15 months, and the median age was 3 months. Three children had previously been treated with laser and failed to achieve significant improvement. The range of ICCs was between 0.91 and 0.98, demonstrating excellent reliability within raters.

**Figure 1:**
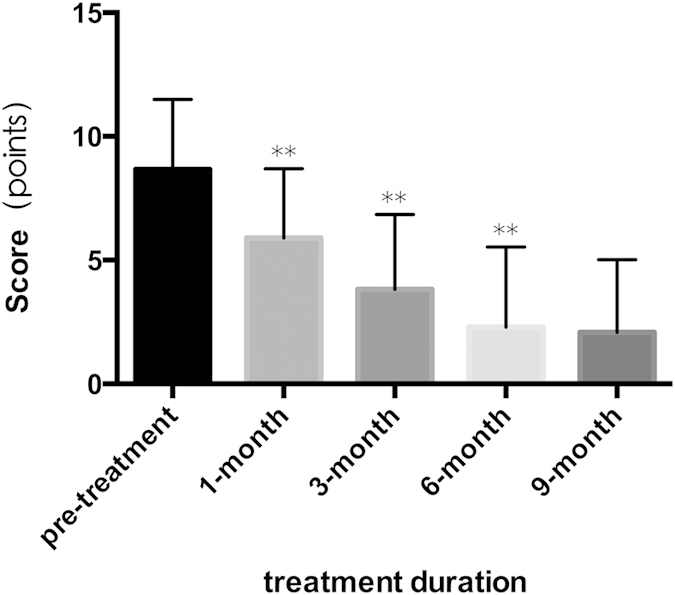
Hemangioma scores before, during and at the end of treatment. Data were expressed as means (95% confidence intervals). n = 89 patients. **indicated p < 0.001 when compared to the previous hemangioma score.

**Figure 2:**
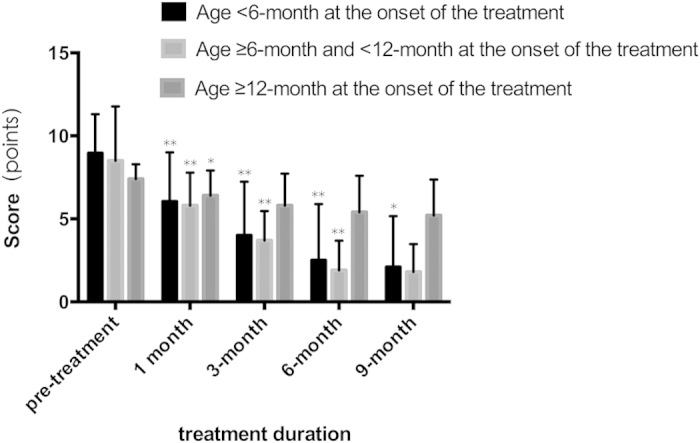
Hemangioma scores of hemangiomas distributed to different ages (months at the beginning of therapy) before, during and at the end of propranolol treatment. Data were expressed as means (95% confidence intervals). *indicated p < 0.05 when compared to the previous hemangioma score, **indicated p < 0.001 when compared to the previous hemangioma score.

Statistical analysis showed that the combined therapy achieved responses in 100% of this case series (89/89). The initial hemangioma score before treatment was 8.67(7.55–9.79), and the score at the end of the treatment was 2.07(0.91–3.23), which indicated that the involution of the hemangiomas was statistically significant (p ≤ 0.001). There was a rapid response to treatment and an immediate statistically significant improvement during the initial 1-month follow-up visit (p ≤ 0.001). During the following clinical evaluation visits, a significant continuous involution of the hemangiomas score was documented until the end of the treatment. When up to 9 months, the improvement compared to the 6-month value was not obvious (p = 0.06) (Fig. 1). Relevant difference exists when distributed according to the patient’s age at the beginning of the combined treatment (Fig. 2). There was a marginally significant improvement observed in children with IH in whom the treatment was started after 12 months of age (p = 0.03).

Of 89 patients, the resolution of hemangioma was complete in 19 children (21.3%), near-complete in 41 children (46.1%), moderate in 18 children (20.2%), fair in 7 children (7.9%), minimal in 4 children (4.5%).

During the following outpatient clinic evaluation visits, few minor side effects were noted: cold extremities (n = 3; 3.4% of all patients), agitation during the night (n = 2; 2.2%) and diarrhoea (n = 2; 2.2%). None of these side effects resulted in a discontinuation of the combined treatment.

The average treatment duration was 6.48 (5.77–7.19) months. After the end of treatment, a rebound growth of the hemangiomas was noted in 1 patient (1.1%). This rebound resulted in a second combined therapy regimen.

**Figure 3:**
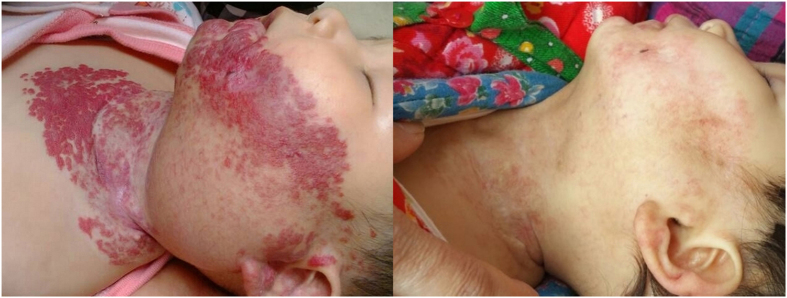
Response of an extensive IH to combined therapy. (**A**), Before treatment, age at starting the treatment was 6.5 months. Hemangioma score was 15. (**B**), End of therapy after 6 months of treatment. Hemangioma score was 1.

**Figure 4:**
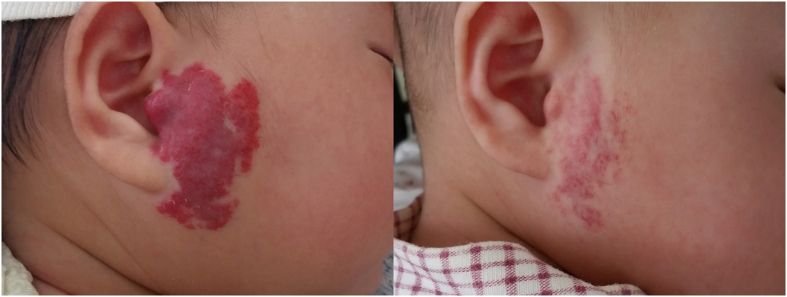
Response of a moderate scalp IH to combined therapy. (**A**), Before treatment, age at starting the treatment was 2.5 months. Hemangioma score was 8. (**B**), End of therapy after 4 months of treatment. Hemangioma score was 1.

Figures 3 and 4 showed patients with compound infantile hemangiomas before and after combined therapy.

## Discussion

Infantile hemangiomas (IH) are the most common benign vascular tumors of infancy. The various risk factors include female gender, prematurity, low birth weight, multiple pregnancies, advanced maternal age and *in vitro* fertilization^1^. Over 60% IH affect the head and neck region. Morphologically, hemangiomas are classified into superficial, deep and compound types. Oral propranolol has become the standard treatment for high-risk and deep IH, whereas topical timolol is commonly used for superficial lesions to minimize systemic side effects. In this study, we introduced a combined therapy consisting of oral propranolol and topical timolol, which is targeting at both superficial and deep components of compound IHs.

In this study, the overall response rate was 100% (89/89), which indicated that the combined therapy could inhibit the growth and promote regression of compound IHs. Different modalities have been reported for treatment of IHs, include laser surgery, cryosurgery, and pharmacotherapy (namely, corticosteroids, vincristine, α-interferon, cyclophosphamide, and propranolol). Their response rates for compound IHs were between 67%–100%^4^,^5^,^6^,^7^,^12^,^13^. Previous case series and observational studies have shown that after the initiation of therapy, a visible change in color and considerable softening of the lesion occurs within 1 month, followed by the growth halt or progressive involution^11^,^14^,^15^,^16^. Our results are consistent with these findings (Fig. 1). The first noticeable effects of the combined therapy were the continuing fading of the color as well as softening of the lesions. The clinically based changes in lesions’ color and firmness have been objectively proven by the statistically significant changes at lesions’ score (P < 0.001). This is related to the β2 inhibitory effect of ß-blockers that decreases the release of the vasodilator transmitters such as nitric oxide. The resulting vasoconstriction of the feeding capillaries is responsible for the early changes in hemangiomas^17^. At 6 months of treatment, the average score was 2.3(1.0–3.6) (p < 0.001 compared to the pre-treatment value), and 16 children (18%) demonstrated complete resolution. The intermediate effects of ß-blocker is due to down regulation of both vascular endothelial growth factors (VEGF) and basic fibroblast growth factors (bFGF) expression in proliferative IH, resulting in inhibition of pro-angiogenic cascade and angiogenesis. The long term effect of ß-blocker is due to apoptosis resulting in regression of hemangiomas^17^. This may be the reason for its use in the post proliferative phase.

Ideally, propranolol should be initiated immediately once the decision is made. In this study, there was significant improvement observed in children younger than 12-month (p < 0.001, compared with the pre-treatment value), while minimal reduction was observed in children older than 12-month (p = 0.03, compare with the pre-treatment value) (Fig. 2). In 59 cases less than 6-month at the beginning of treatment in the present study, a considerable shortening of the natural course of IHs (55/59) had been achieved for those lesions at proliferative phase. This finding was in accordance with previous studies: A recent systematic review (including 1264 children in 41 studies) showed that oral propranolol was initiated at a mean age of 6.6 months (range from 3 days to 10 years)^18^. Other researchers did not observe any improvement in children with IH in whom the propranolol was started after 9 months of age^16^. Previously published studies also indicated that early treatment with oral propranolol, particularly when started during the proliferative phase, has been shown to be associated with better long-term outcomes^19^,^20^,^21^. Although no large prospective studies confirm that early start of propranolol therapy results in better outcomes, it is always better to start the treatment as early as possible during rapid proliferation of the tumor.

The duration of therapy depends on the extent of involvement and the sensitivity to the treatment. A systematic review of 1264 children in 41 studies found that the propranolol was administered for an average duration of 6.4 months (range 1 weeks to 15 months)^18^. In a recent study on propranolol treatment for complicated IH, the authors have used the drug for a mean duration of 10.7 months^22^. In this study, the mean duration of treatment was 6.48 (5.77–7.19) months, which indicated that the combined therapy may shorten the treatment duration. However, one child (2-month old) had relapse after cessation of 6-month combined therapy. This is consistent to the previous studies which suggested that the optimal propranolol treatment must at least cover the entire proliferative phase of compound IHs^4^,^11^,^22^. As for larger and deeper IHs, due to their protracted late proliferative phase, the treatment could extending to the age of 12 months^23^.

The reported side effects of ß-blocker include hypotension, bradycardia, hypoglycaemia, pulmonary symptoms, sleep disturbances, somnolence, cold extremities and gastro-intestinal (GI) complaints. These adverse reactions are reversible, dose-dependent, and not serious^24^. In a systematic review^18^ of 39 studies in 1189 children, 371 cases (31.2%) had adverse reactions. The most common adverse event was sleep changes in 136 cases (11.4%), followed by acrocyanosis in 61 children (5.1%). There were 41 reports (3.4%) of GI problem like diarrhoea and gastroesophageal reflux. Respiratory adverse events were seen in 35 patients (2.9%). Hypotension and bradycardia were seen in 39 (3.3%) and 8 cases (0.7%), respectively. Hypoglycaemia was reported only in 4 cases (0.3%). In this study, seven children (7.8%) developed side effects: three children (3.4%) with cold extremities, two children (2.2%) with agitation during the night and 2 children (2.2%) with diarrhoea. The incidences of total and subcategory side effects were lower than the data mentioned above, which indicated that the combined therapy could be a safe choice for compound IHs. No patient had serious side effects leading to discontinuation of the combined therapy. Oral propranolol was administered strictly after feeding, and parents are advised to avoid long periods of fasting, this might be the reasons that hypoglycaemia didn’t occur in our series.

Currently there is no universally accepted protocol for initiation, dosage and monitoring of topical timolol treatment. As timolol is 4–10 times as potent as propranolol^25^, the overall dose of concomitant use of oral propranolol and topical timolol should be taken into consideration. In the recent consensus conference report on propranolol treatment for hemangioma, a target dose of 1–3 mg/kg has been recommended^24^, and the average treatment dose of propranolol was 2.1 mg/kg/day^24^. In this study, the dosage of propranolol was 2 mg/kg/day, while the dosage of timolol maleate 0.5% gel (5 mg/mL) was 8.33 mg per day (50 ml gel for 1 month, dosage for a moderate IH). In general, the percutaneous systemic absorption of most topical drugs occupies only a small percentage of total drug amounts. Using a revised and probably still overly cautious estimate of the systemic bioavailability of 5%–10% topical timolol gel-forming solution would yield equivalent oral propranolol doses of only 0.2 to 1.0 mg, or 0.05 to 0.25 mg/kg for a 4-kg infant (0.03–0.17 mg/kg for a 6-kg infant, i.e., a 3-month-old in the 50th percentile for weight)^26^. These estimates suggest far less need for caution, and systemic side effects of topical timolol are unlikely to occur. However, until larger clinical trials are completed and the safety is fully established, the combined therapy should be used with caution, especially in preterm infants, IH with large body surface areas.

IHs are the most common benign vascular tumors in children. They are a cause of parental discomfort and anxiety and need to be carefully assessed from the treatment point of view. In this study, oral propranolol combined with topical timolol treatment was proven to be very effective and safe for compound IHs, this may offer a better first line treatment. However, future prospective trials involving a larger number of patients and a longer follow-up period should be addressed to determine whether this combined therapy fulfils its therapeutic promise. Anticipatory guidance and appropriate monitoring protocol are important for the safe use of the combined therapy.

## Conclusion

This retrospective study revealed that oral propranolol combined with topical timolol treatment is very effective and safe for compound IHs, which can be used as a first line treatment. Future prospective trials involving a larger number of patients and a longer follow-up period should be addressed to determine whether this combined therapy fulfils its therapeutic promise.

## Additional Information

**How to cite this article**: Jing Ge. *et al*. Oral propranolol combined with topical timolol for compound infantile hemangiomas: a retrospective study. *Sci. Rep.* **6**, 19765; doi: 10.1038/srep19765 (2016).

## Figures and Tables

**Table 1:** Hemangioma score.

**Component**	**Quality**	**Score**
(1) Color of the hemangioma	Bright red	2
	Pale	1
	Skin color	0
(2) Surface consistency	Markedly raised	2
	Raised	1
	Flat	0
(3) Firmness	Firm	2
	Softer	1
	Not firm or much softer	0
(4) Depth (if ultrasound is performed; otherwise ‘0’)	Maximal (90%–100%)	2
	Less (50%–89%)	1
	No depth or much less deep (<50%)	0
(5) Organ involvement	Functional limitation	7
	Impending functional limitation	4
	None	0
Total score		0–15

**Table 2:** Patients’ demographic data and lesions’ data before treatment.

**Category**	**Subcategory**	**Number**	**Percentage**
Sex	female	53	59.6%
	male	36	40.4%
Age	<6 mo	59	66.3%
	≥6 mo and <12 mo	23	25.8%
	≥12 mo	7	7.9%
Birth	on-term	79	88.8%
	preterm	10	11.2%
Region	focal	66	74.2%
	segmental	23	25.8%
Site of IH	face	40	43.0%
	periorbital and eyelids	15	16.1%
	nose	14	15.1%
	scalp	11	11.8%
	parotid region	10	10.8%
	ear	3	3.2%
Presenting complaints	Cosmetic disfigurement	76	85.4%
	Feeding problems	9	10.1%
	Visual obstruction	5	5.6%
	Nasal obstruction	3	3.4%
